# A Case of Atrial Standstill Misdiagnosed as Atrial Fibrillation: Diagnostic Insights and Clinical Implications

**DOI:** 10.1016/j.jaccas.2025.103873

**Published:** 2025-05-14

**Authors:** Zhengbo Wu, Yang Wu, Yang Liu, Jiqiang Hu

**Affiliations:** a Graduate School, Beijing University of Chinese Medicine, Beijing, China; b Department of Cardiology, Dongfang Hospital, Beijing University of Chinese Medicine, Beijing, China

**Keywords:** atrial cardiomyopathy, atrial fibrillation, atrial standstill, case report, giant atria

## Abstract

**Background:**

Atrial standstill (AS), a rare and diagnostically challenging cardiac disorder with total absence of atrial electrical and mechanical activity, often presents variably, mimicking atrial fibrillation (AF) in electrocardiogram (ECG). This report details a case of AS initially misdiagnosed as AF.

**Case Presentation:**

A 65-year-old Chinese female had 5-year intermittent palpitations. Prehospital ECG showed paroxysmal AF, and echocardiogram showed a giant atrium. A single-chamber pacemaker (ventricular pacing, ventricular sensing, inhibited) was implanted 6 months ago due to long ventricular intervals. During this hospitalization, AS was confirmed using electrophysiological examination, altering the treatment approach and prognosis.

**Conclusions:**

AS is complex and overlooked. Early recognition and proper management are crucial. Accurate diagnosis via echocardiogram and electrophysiological testing is vital for at-risk patients. This case highlights the need for precise test interpretation and comprehensive evaluation for optimal patient care. AS is an infrequently encountered arrhythmia that eludes clear diagnosis using surface ECG. It is characterized by complete atrial electrical and mechanical inactivity, bradycardia, atrioventricular junction escape rhythm, and absence of P waves. Despite its rarity, patients may present with palpitations, syncope, or stroke, warranting diagnostic attention. Acquired AS can result from myocardial infiltrative diseases like amyloidosis, sarcoidosis, and hemochromatosis, as well as infections (viral myocarditis), autoimmune disorders, and certain medications. Here, we present a case of AS potentially due to long-term AF.

## Graphical Abstract

**Figure:**
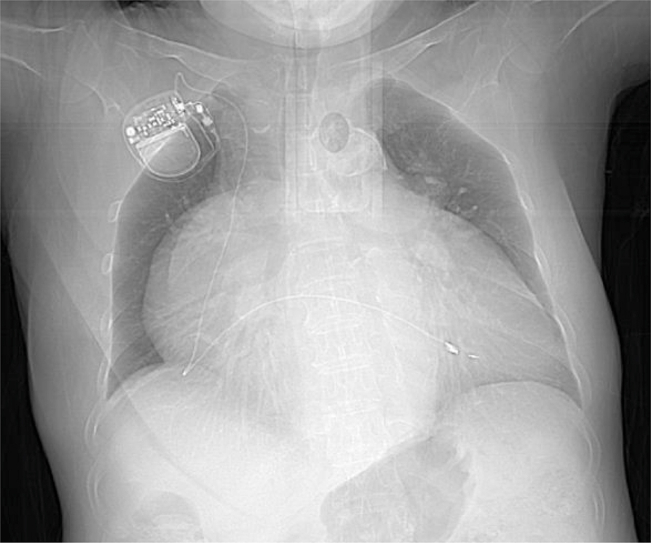

## Case Presentation

**Figure 1:**
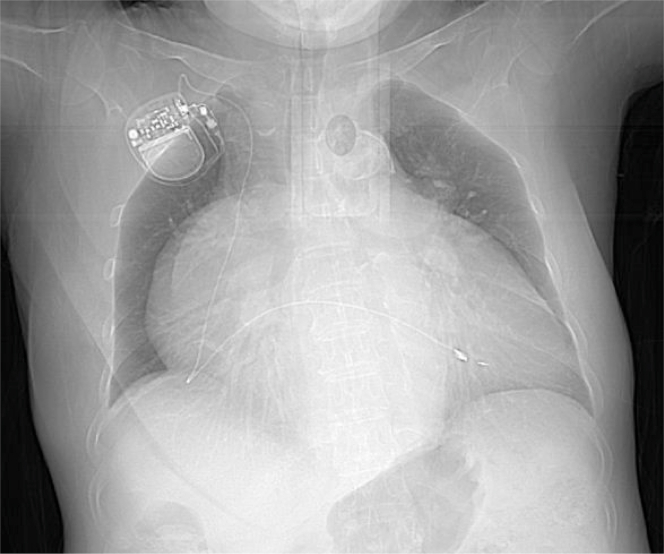
Chest Radiograph The chest radiograph shows that the patient has a huge heart.

**Figure 2:**
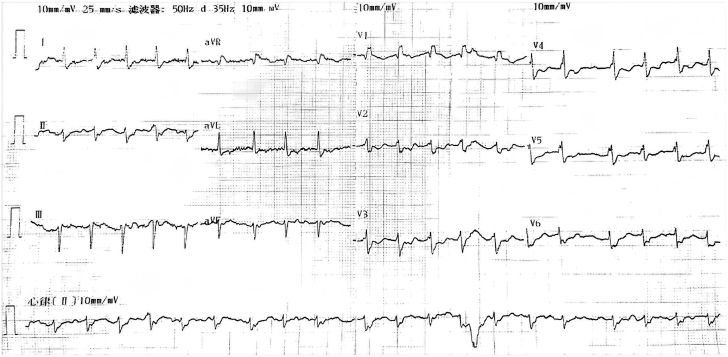
Electrocardiogram The electrocardiogram obtained upon the patient’s admission suggested suspected atrial fibrillation.

A 65-year-old Chinese female endured intermittent palpitations for 5 years. The prehospital electrocardiogram (ECG) indicated paroxysmal atrial fibrillation (AF), and chest radiograph revealed a giant heart (Figure 1). One week before current hospitalization, she presented with frequent palpitations and was diagnosed with AF via ECG (Figure 2). She was then admitted to the Cardiovascular Department.

**Take-Home Messages**

• The misdiagnosis of AS as AF emphasizes the complexity and subtlety of cardiac arrhythmia diagnosis, urging clinicians to be vigilant.
• For patients with AS, accurate diagnosis using electrophysiological examination and timely implementation of treatment measures such as ventricular pacing are crucial for improving patient prognosis and the quality of medical care.

**Figure 3:**
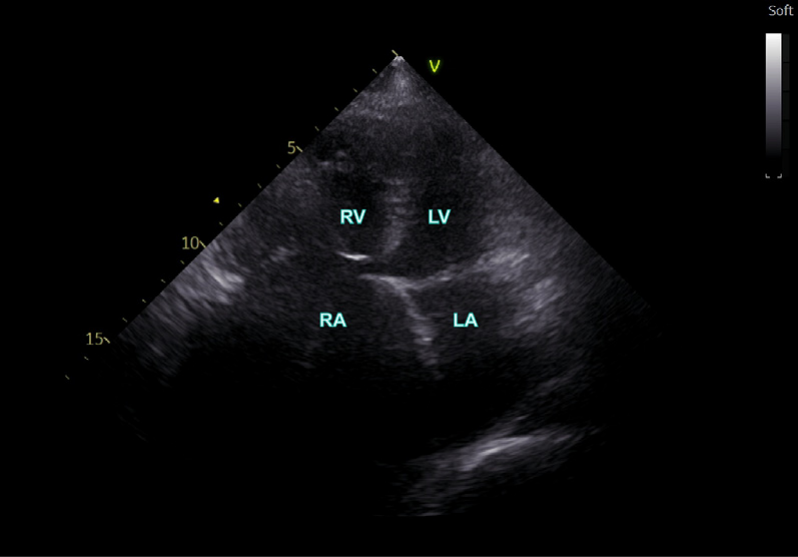
Echocardiogram The echocardiogram demonstrated a significantly enlarged atrium. Specifically, the inner diameter of the left atrium measured 51 mm while that of the right atrium was 71 mm. LA = left atrium; LV = left ventricle; RA = right atrium; RV = right ventricle.

On admission, her blood pressure was 113/76 mm Hg. Lungs were clear, bilateral heart border enlarged, and a grade 3/6 systolic murmur of the tricuspid valve was heard. The admission ECG showed no P waves, no distinct f waves, and supraventricular QRS waves. The bedside echocardiogram demonstrated a giant atrium with a left atrial inner diameter of 51 mm and a right atrial inner diameter of 71 mm (Figure 3), along with moderate tricuspid regurgitation and no A peak of the mitral valve.

Six months ago, a single-chamber pacemaker (ventricular pacing, ventricular sensing, inhibited) was implanted in the right ventricle due to significant bradycardia and long ventricular intervals. The patient was on dabigatran (110 mg, twice a day) for stroke prevention.

**Figure 4:**
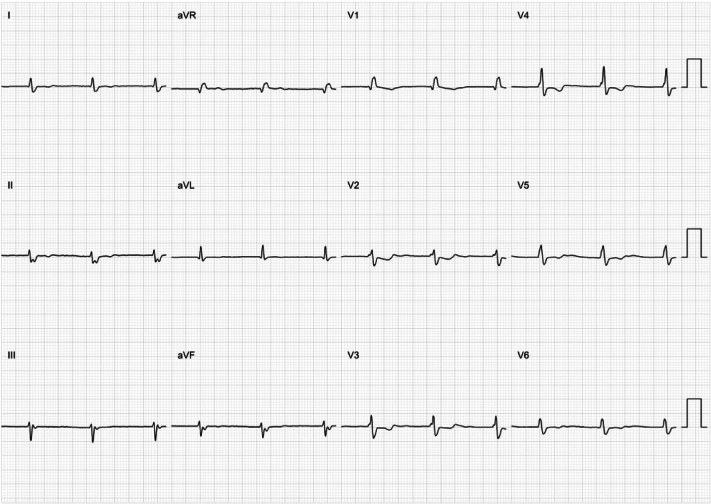
Electrophysiological Examination The electrophysiological examination revealed that the surface ECG presented junctional rhythm, lacking distinct P-waves. Abbreviation as in Figure 2.

**Figure 5:**
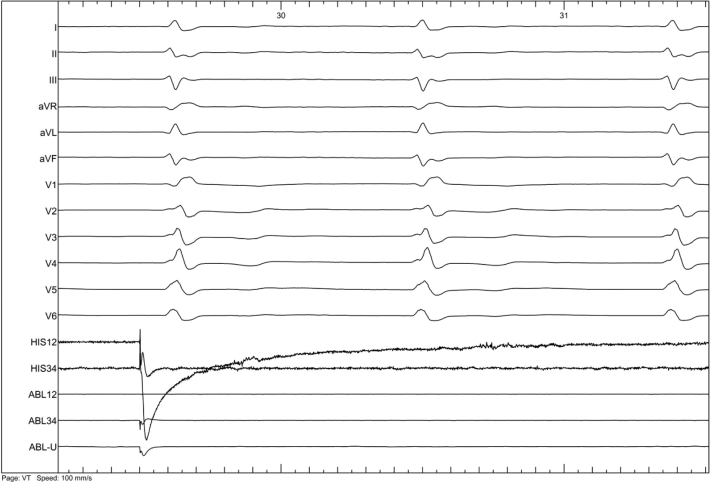
Pacing the Right Atrium When pacing the right atrium with a voltage of 10 V (2.0 ms), no capture was observed. This finding indicated the absence of atrial electrical activity, confirming the diagnosis of AS. AS = atrial standstill.

Based on clinical evidence, the main differential diagnosis was atrial standstill (AS) induced by atrial cardiomyopathy. After obtaining informed consent, an electrophysiological examination was performed. The ECG showed junctional rhythm without obvious P wave (Figure 4). During the electrophysiological examination, pacing tests were meticulously carried out at multiple key atrial sites, including the free wall of the right atrium, the right atrial appendage, the interatrial septum, and the coronary sinus. To ensure comprehensive assessment, the pacing voltage was elevated to its maximum level (range: 5-10 V, pulse width: 1.0-2.0 ms). In every instance, the atrium failed to be captured, verifying that the atrial electrical activity had vanished and that it was AS (Figure 5). The previous AF diagnosis was revised.

Written informed consent was obtained from the patient for publication of this case report and any accompanying images.

## Discussion

In this case, the enlarged atrium and suspected cardiomyopathy disrupted the atrial electrophysiological environment. AS occurs due to the complete absence of atrial electrical and mechanical activity, often related to atrial myocardial cell and conduction system dysfunction, caused by advanced cardiac diseases, myocardial fibrosis, or genetic disorders.^1^

Differentiating AS from AF is crucial. Their ECG manifestations differ, with AF having chaotic atrial electrical activity and AS lacking it. Clinical manifestations and potential complications also vary, with AF associated with palpitations and thromboembolic risks, and AS with decreased cardiac output and junctional arrhythmias. The diagnosis of AS needs the following criteria. First, on the electrocardiogram, the P-waves should be completely absent. Second, cardiac electrophysiological examination must confirm the absence of any spontaneous electrical activity within the atrium. Third, other conditions that could potentially result in the disappearance of P-waves, including hyperkalemia and severe sinoatrial node dysfunction, need to be meticulously excluded.

Genetically, AS may be linked to SCN5A or HCN4 variants.^2^ Patients at risk of thromboembolic events due to lack of atrial pacing require anticoagulation or left atrial appendage closure. Accurate diagnosis of AS is essential for proper management and prognosis. Echocardiogram helps identify structural and functional atrial changes, and advanced electrophysiological studies clarify electrical abnormalities.

Treatment for AS depends on the cause and patient’s condition. Permanent AS may necessitate pacing therapy,^3^ with close monitoring and follow up to assess treatment effectiveness and detect complications. Left atrial appendage occlusion mainly prevents thromboembolic events. The atrium’s weakened activity leaves the left atrial appendage, with its special structure, a high-risk thrombus site due to slow, eddy-forming blood flow and hypercoagulability. The procedure removes this thrombus source, reducing stroke risks. Because AS is rare, there was scarce relevant literature and no reliable evidence yet.

This case underscores the critical significance of comprehensive and precise diagnosis. The initial misdiagnosis and the subsequent accurate diagnosis vividly demonstrate the imperative necessity for continuous monitoring of ECG and meticulous interpretation of echocardiogram. The discovery of the giant atrium and the electrophysiological findings ultimately paved the way for the correct diagnosis, which serves as a valuable lesson in the field of cardiology, emphasizing the importance of thorough examinations and vigilant monitoring in ensuring accurate disease identification and appropriate patient management.

Continuous monitoring and follow-up are also significant. The 10-month follow-up spanning from April 2024 to January 2025 not only attests to the effectiveness of the treatment but also accentuates the significance of long-term management. Overall, this case enhances clinicians’ understanding of cardiac arrhythmia differential diagnosis and treatment strategies for AS, improving the quality of patient care and optimizing health outcomes.

## Conclusions

AS is a complex and frequently overlooked cardiac condition. Timely recognition and appropriate management, such as pacemaker implantation, are crucial for AS patients. For the ill at risk, achieving an accurate diagnosis through echocardiogram and electrophysiological examination is of paramount importance. This not only empowers medical professionals to make well-informed treatment decisions but also plays a pivotal role in enhancing the overall quality of patient care, ultimately leading to better health outcomes.

### Data availability

All data relevant to this case report are included within the article. Any additional data not presented in the article can be made available on request to the corresponding author.

## Funding Support and Author Disclosures

This research was supported by Capital Health Research and Development of Special Fund, China (2020-2-4203). The authors have reported that they have no relationships relevant to the contents of this paper to disclose.
